# High-Efficiency Solar Steam Generation Using a Biochar-Modified Sponge Evaporator Derived from Turkish Coffee Waste

**DOI:** 10.1021/acsomega.5c06761

**Published:** 2025-10-01

**Authors:** Şeyda Sefa, Kader Dağcı Kıranşan, Ezgi Topçu

**Affiliations:** Department of Chemistry, Science Faculty, 37503 Atatürk University, Erzurum 25240, Türkiye

## Abstract

Solar seawater desalination represents a sustainable approach to addressing the global freshwater shortage. Nevertheless, the high cost of photothermal materials remains a significant obstacle to the large-scale production of solar steam generation (SSG) systems. Herein, we developed a cost-effective photothermal evaporator (CTCW-S) by integrating carbonized Turkish coffee waste (CTCW) with a commercially available, hydrophilic, self-floating cellulosic sponge (S). The CTCW was obtained through pyrolysis of collected coffee waste, yielding a porous carbonaceous material with strong photothermal properties. The combination of the sponge’s rapid water absorption capability and the adequate solar absorption efficiency of the CTCW enabled the resulting material to generate efficient steam. Under one solar illumination, CTCW-S demonstrated a water evaporation rate of 1.63 kg m^–2^ h^–1^ and a solar-to-vapor conversion efficiency of 81%. These results underscore the potential of CTCW-S as a cost-effective and scalable solution for water purification and seawater desalination. Utilizing biomass-derived waste in photothermal materials presents a novel and sustainable approach to addressing global freshwater scarcity.

## Graphical abstract

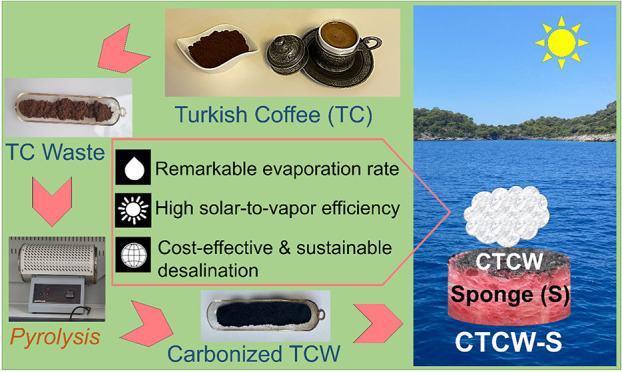

## 1 Introduction

The diminishing availability of clean water constitutes a critical threat to human survival and the achievement of sustainable development. Currently, brackish water purification and municipal wastewater, and desalination of seawater are considered the primary methods to reduce stress on water resources.^1–4^ In response to increasing water scarcity, there has been a substantial expansion in the deployment of seawater desalination plants in water-stressed regions to augment freshwater supply.^5–8^ Among the available technologies, multistage distillation and reverse osmosis remain the predominant methods employed in thermal and membrane-based desalination, respectively, collectively comprising the majority of the global desalination market.^9–11^ Nevertheless, their widespread implementation is constrained by the high energy demands associated with thermal and electrical power consumption, as well as the need for extensive infrastructure, thereby limiting their potential to deliver safe and sustainable drinking water on a large scale.^12–14^ At this point, solar energy emerges as an inexhaustible renewable green energy source offering a sustainable and environmentally friendly alternative compared to other energy sources.^15–17^ Thus, solar steam generation (SSG) using photothermal materials is a green, low-cost, and efficient process for solving the water crisis with sustainable energy. From the standpoint of energy efficiency, the optimal design of a water evaporation system should integrate the following key components: (1) photothermal materials capable of efficiently converting solar energy into thermal energy; (2) structural configurations that both maximize the evaporation surface area and facilitate continuous water transport to the heated interface; and (3) thermal insulation layers that minimize heat dissipation to the underlying water reservoir and surrounding environment.

Considerable advancements have been achieved in the development of diverse photothermal materials^18–20^ and supporting substrates^21,22^ for SSG. Photothermal materials encompass a broad range of classes, including carbon-based compounds, plasmonic metal nanoparticles, semiconductors, and organic polymers.^23^ Common hydrophilic substrates comprise materials such as hydrogels and hydrophilic sponges.^24–26^ Notably, ongoing research in photothermal materials has facilitated the application of highly efficient solar-absorbing nanostructures, including gold (Au), silver (Ag), and palladium (Pd) nanoparticles.^27^ However, high-cost raw material requirements, complex production processes, and low stability prevent their use in practical applications. Therefore, the demand for the development of cost-effective and environmentally benign materials for water treatment remains ongoing.^28,29^

Recent advances in photothermal active materials have accelerated the development of interfacial SSG by combining high solar absorption, efficient heat localization, and engineered water-transport pathways. Biomass-derived carbonaceous materials are particularly attractive because they are low-cost and sustainable and readily yield porous, broadband-absorbing structures after simple carbonization procedures. For instance, Jiang et al. demonstrated a three-dimensional robust solar evaporator derived from biomass, achieving efficient steam generation, water purification, and salt-resistant desalination.^30^ Hu et al. reported carbonized biomass from agricultural residues with high photothermal conversion efficiency, providing a scalable and economically viable approach for interfacial SSG.^31^ More recently, Karothiya et al. reviewed recent advancements in biomass-based photothermal materials, highlighting their tunable thermal properties, broadband absorption, and enhanced water-transport performance for sustainable solar-driven evaporation.^32^ Collectively, these studies illustrate how emerging biomass-derived photothermal materials, including biochar obtained from various biomass sources, can deliver effective and durable photothermal layers suitable for practical desalination and wastewater treatment while motivating further optimization of structural design, salt-management strategies, and life-cycle energy analyses.

Biochar, a carbon-rich solid material composed of carbon and mineral constituents, is produced through thermochemical conversion processes, such as combustion, hydrothermal carbonization, gasification, torrefaction, and pyrolysis, carried out under limited or absent oxygen conditions using various biomass feedstocks. Although wood has been used as biomass since ancient times, biochar can be produced from corn cobs, rice husks, peanuts, tree shells, processed paper waste, etc.^33–35^ Among these, coffee waste is a low-cost and abundant biomass source.^36–38^

There is a large coffee consumption volume in Turkey as in the world. Turkish coffee (TC) is regarded as part of Turkish cultural heritage, and this element was inscribed in 2013 on the UNESCO Intangible Cultural Heritage of Humanity. TC, among others, is the most consumed and favored coffee by Turkish society. Thus, TC waste, a biomass source, is formed through this huge consumption.

The adverse environmental and human health impacts associated with waste materials have intensified research efforts aimed at converting waste into value-added products. In this context, the synthesis of biochar for environmental remediation represents a prominent “waste-to-wealth” strategy, offering a dual benefit by facilitating both effective waste management and the sustainable utilization of biomass residues.^39^ For an ideal solution to purify polluted water, raw material cost and environmental impact should be taken into consideration. Therefore, carbonized natural materials, especially biomass waste, are extremely important in solar water purification due to providing an opportunity for recycling. Nevertheless, studies on the preparation of photothermal material for SSG through the utilization of waste biomass are limited.

In this work, we fabricated a photothermal material (CTCW-S) by modifying a hydrophilic self-floating commercial sponge (S) with carbonized TC waste (CTCW) for SSG. First, a pyrolysis process was applied to the TC waste collected to prepare a photothermal carbonized structure. Subsequently, an inexpensive cellulosic sponge was modified with the resulting porous-biomass-derived carbonaceous structure to obtain a novel solar steam generator. The easy and rapid water absorption ability of the S, combined with the superior light absorption and high solar-vapor conversion of the CTCW, showed superior solar steam generation performance. The findings exhibited that CTCW-S, with a water evaporation rate of 1.63 kg m^–2^ h^–1^ and a solar-to-vapor conversion efficiency of 81% under one sun illumination, is a promising low-cost solar evaporator for solar desalination. CTCW-S will bring a new perspective to the utilization of waste-based materials for SSG and provide a viable alternative to addressing freshwater scarcity.

## 2 Experimental Section

### 2.1 Materials

TC is drunk at home and in the office daily. TC waste (TCW) was obtained by collecting the coffee grounds left at the bottom of the coffee cups (Figure S1). The commercial natural cellulosic bath sponge (Wee Baby) was supplied by a local supplier in Türkiye. NaCl, MgCl_2_, KCl, CaCl_2_, Cr­(NO_3_)_3_, Pb­(NO_3_)_2_, Cu­(NO_3_)_2_, Ni­(NO_3_)_2_, HCl, NaOH, methylene blue (MB), and poly­(vinyl alcohol) (PVA) were purchased from Sigma-Aldrich.

### 2.2 Preparation of CTCW

TCW was washed and dried at 60 °C. Then it was loaded into a tube furnace and heated in an argon atmosphere at different temperatures (300, 400, 500, and 600 °C) for 2 h (Figure S1). The CTCWs were named CTCW-300, CTCW-400, CTCW-500, and CTCW-600.

### 2.3 Preparation of Solar Evaporator: CTCW-S

To prepare the photothermal material, first, a commercial natural cellulosic sponge (S) was cut into a cylinder with about 4 cm in diameter (Figure S2). Subsequently, a poly­(vinyl alcohol) (PVA) solution (0.05g/mL) was prepared, and the circular surface of the sponge was dipped into this solution. This surface of S was homogeneously coated with the optimized amount of CTCW. At the surface, the PVA solution and CTCW were mixed and, thus, fixed on the sponge surface (Figure S2).

### 2.4 Characterization

The surface morphology and elemental composition of the samples were characterized by using a ZEISS SIGMA 300 field-emission scanning electron microscope (FESEM) equipped with an energy-dispersive X-ray spectrometer (EDS) (Munich, Germany). Raman spectra of CTCW were obtained using a WITec α 300R micro-Raman spectrometer (Ulm, Germany) with a 532 nm excitation laser. The crystalline structures of the samples were analyzed by powder X-ray diffraction (XRD) using a Rigaku MiniFlex diffractometer (Neu-Isenburg, Germany) with Cu Kα radiation (λ = 1.5406 Å). X-ray photoelectron spectroscopy (XPS) measurements were conducted by using a Spect-Flex spectrometer (Berlin, Germany) with monochromatic Al Kα radiation (1486.71 eV). The optical properties in the ultraviolet–visible-near-infrared (UV–vis-NIR) range were evaluated by using a Shimadzu 3101PC spectrophotometer. Ion concentrations were quantified via inductively coupled plasma mass spectrometry (ICP-MS, Agilent 7800). Simulated solar irradiation, ranging from 1 to 10 solar irradiations (1–10 kW·m^–2^), was provided by an OptaSense OPT-S500F photocatalytic xenon light source. Surface temperatures of the CTCW-S samples under irradiation were monitored by using a FLIR T620 infrared (IR) thermal imaging camera (FLIR Systems, Inc., USA).

### 2.5 Solar Evaporation Experiments

Indoor evaporation experiments were conducted under controlled conditions at a temperature of 25  ±  2 °C and a relative humidity of approximately 50%. The photothermal material, CTCW-S, had a diameter of 4 cm and a height of 1.5 cm. To minimize thermal losses to the environment, a vacuum-insulated, double-walled glass container with a volume of 40 mL was employed. A solar simulator equipped with a xenon lamp was used to provide variable light intensities ranging from 1 to 10 suns (1–10 kW·m^–2^), mimicking solar irradiation. The surface temperature of CTCW-S during irradiation was continuously monitored using an infrared (IR) thermal imaging camera. Water mass loss was measured using a high-precision electronic balance (Shimadzu AUW220D, accuracy: 0.00001 g). The evaporation rate was calculated from the slope of the mass change curve. For comparative analysis, outdoor evaporation experiments were also conducted in the courtyard of the Department of Chemistry at Atatürk University.

## 3 Results and Discussion

### 3.1 Characterization of CTCW

Biomass-derived carbonaceous materials are considered highly suitable for SSG applications owing to their cost-effectiveness and advantageous physicochemical properties, including low toxicity, broad-spectrum solar absorption, and low thermal conductivity. A scalable and facile carbonization process was applied to Turkish coffee grounds to obtain a CTCW. Figure S3 shows digital photographs of carbonized samples obtained after pyrolysis at different temperatures. To better explain the thermal behavior of TCW, Figure S4 presents the thermogravimetric (TG) and derivative thermogravimetric (DTG) curves, reflecting their thermal stability. The first decomposition step for TCW occurred between 35 and 180 °C due to the removal of moisture and volatile compounds. The TCW biomass showed maximum weight losses of ∼58% and ∼32% at around 316 and 419 °C, respectively. When the temperature exceeded 500 °C, almost complete pyrolysis was achieved, leaving only a minimal solid residue. Accordingly, pyrolysis temperatures of 400, 500, and 600 °C were selected for the optimization studies. Figure 1a–c presents the FESEM images of CTCW-400, CTCW-500, and CTCW-600. Although the samples show similar morphology in each FESEM image, it can be seen that the gaps and roughness are formed in the structure as the pyrolysis temperature increases. Such a structure provides more surface area, which is advantageous for photothermal material since it facilitates water evaporation. CTCW-500 has a rougher and gapped surface compared to other samples. The EDS spectrum was performed for the elemental analysis, and the composition of CTCW-500 was confirmed by the presence of C (70.7%) and O (29.3%), demonstrating a successful preparation of CTCW (inset of Figure 1b).

**Figure 1:**
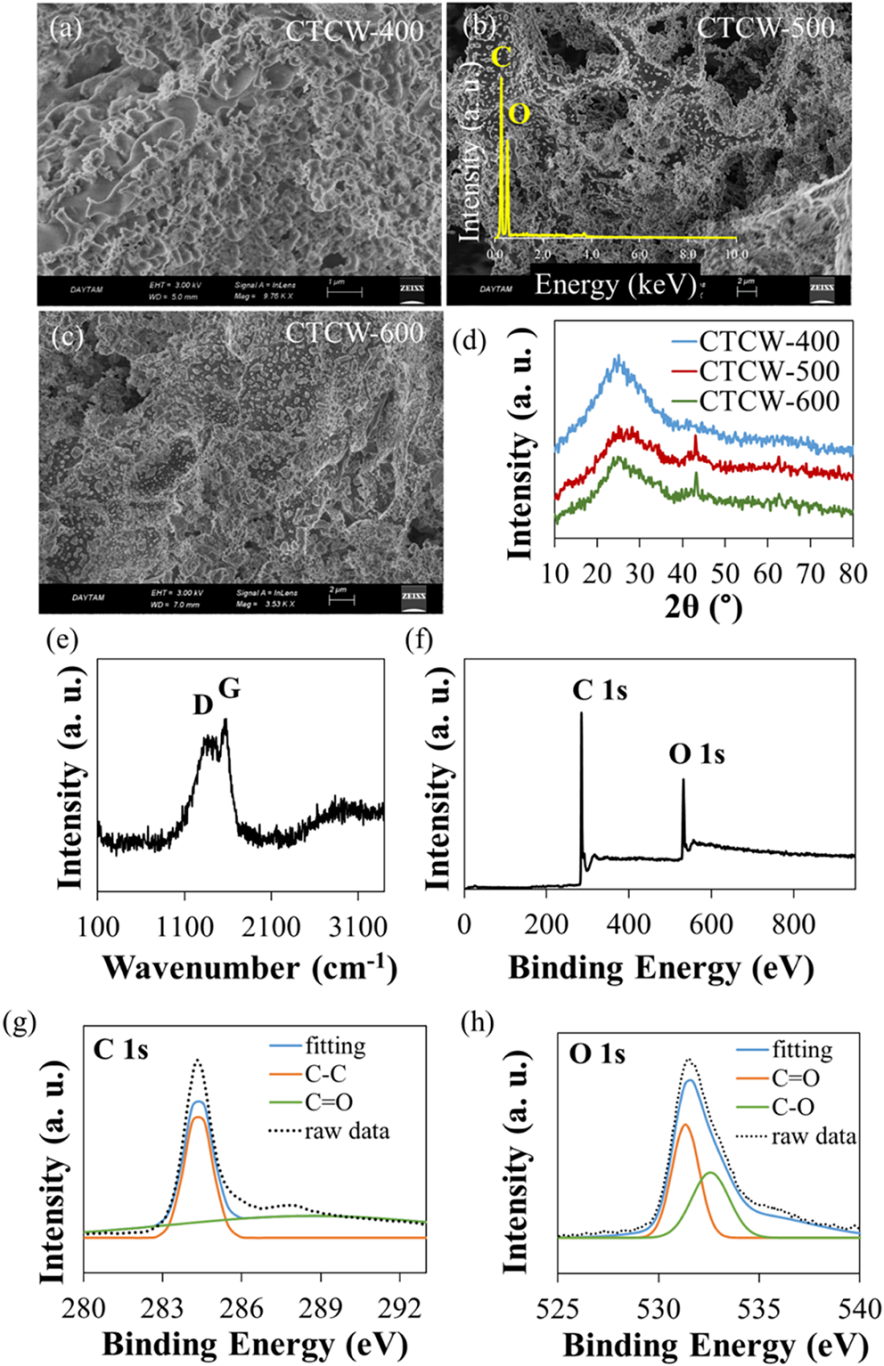
(a–c) FESEM image of CTCW-400, CTCW-500, and CTCW-600. Inset: EDS spectrum of CTCW-500. (d) XRD data of CTCW-400, CTCW-500, and CTCW-600. (e) Raman and (f) XPS survey spectrum of CTCW-500. Deconvoluted (g) C 1s and (h) O 1s XPS spectra of CTCW-500.

Figure S5 presents the N_2_ adsorption–desorption isotherms and pore size distributions of CTCW-300, CTCW-400, CTCW-500, and CTCW-600. The specific surface areas of the samples were calculated using the Brunauer–Emmett–Teller (BET) method, whereas the pore volumes and average pore sizes were determined according to the Barrett–Joyner–Halenda (BJH) method. For the CTCW samples obtained at different pyrolysis temperatures, the pore sizes were found to vary with increasing temperature: approximately 2 nm at 300 °C, around 2–22 nm at 400 and 500 °C, and within the range of 2–50 nm at 600 °C. This evolution indicates that the increase in temperature promotes the decomposition of organic structures, leading to enhanced gas release and consequently a transition in pore structure from the microporous to the mesoporous domain. The BET surface areas of CTCW-300, CTCW-400, CTCW-500, and CTCW-600 were calculated to be 95.2, 110.8, 119.7, and 127.2 m^2^ g^–1^, respectively. Based on these results, 500 °C was selected as the pyrolysis temperature for preparing biochar as a photothermal material in SSG applications, as it provides an appropriate balance between the surface area and pore structure.

The crystal structure of carbonized samples was characterized through XRD. In Figure 1d, XRD data of CTCW-500 shows a peak at 24°, corresponding to the (002) graphitic carbon plane, besides a broad peak at about 43°, associating diffraction of sp2 hybridized carbon honeycomb structures (100).^40,41^ When the pyrolysis temperature increased from 400 to 500 °C, the graphitic carbon peak shifted to the higher 2θ values, while the intensity of (100) diffraction exhibited an increment. This slight shift is related to the decrease in the interlayer distance. According to Bragg’s Law, the interlayer distances (*d*_002_) of CTCW-400, CTCW-500, and CTCW-600 were calculated as 0.350, 0.332, and 0.353 nm, respectively. CTCW-500 has a lower *d*_002_ and, therefore, a more compact structure, which can provide a benefit in heat conduction. Figure 1e shows the Raman spectrum of CTCW-500. The two characteristic peaks observed at approximately 1563 and 1395 cm^–1^ correspond to the E_2g_ phonon mode of sp^2^-hybridized C=C bonds in the two-dimensional hexagonal carbon lattice (G band) and the vibrational modes of sp^3^-hybridized carbon atoms associated with structural disorder in graphite (D band), respectively.^42^ The intensity ratio (*I*_G_/*I*_D_) depends on the graphitization degree and was estimated as 0.88 for CTCW-500, indicating structural defects in the carbonized structure. The Raman broad peak at about 2920 cm^–1^ is for a D + G combination mode and originates from a disorder.^43^ XPS further investigated the chemical states of CTCW-500. Figure 1f demonstrates the presence of 68% C and 32% O elements, indicating the successful preparation of CTCW. C 1s at 286.7 eV and O 1s at 533.1 eV exhibit deconvoluted peaks in Figure 1g,h, which are assigned to C=O, C–C (for C 1s), and C=O, C–O (for O 1s) chemical bonds, respectively.^44^ Considering all of these results, 500 °C was optimized for the pyrolysis temperature of CTCW preparation, and photothermal material (henceforth called CTCW-S) fabricated with CTCW-500 was used in SSG studies.

Figure S6 presents the UV–vis–NIR absorption spectra of CTCW. The absorption peak observed at 233 nm is attributed to the π–π* electronic transition of C–C bonds, consistent with previously reported findings.^44^ In addition to the UV region, CTCW has absorption features in the visible and particularly NIR (in the range 840–1100 nm), demonstrating that CTCW absorbs sunlight in a wide wavelength range as a photothermal material.

Efficient and rapid water absorption throughout the material matrix is a critical parameter influencing its performance in SSG applications. To test the water absorption of the natural cellulosic sponge, S (with a 4 cm diameter and a 1.5 cm height) was kept in water for 1 min, and then the weight of the sponge was compared with the initial weight (Figure S7). The dry and wet weights of S are 1.5625 and 20.9030 g, respectively. There is about a 13 times difference between dry and wet S, demonstrating that the natural sponge absorbs water about 12 times its weight.

The morphology of the bare S and the CTCW-S was characterized through FESEM. Figure 2a,b shows that the sponge has a rough layered structure consisting of different-sized pores. These disordered pores are beneficial for water absorption and evaporation. CTCW is coated homogeneously inside the pores (Figure 2c,d), and the roughness of the surface increased due to the stacked CTCW, which contributes to a higher solar absorption for evaporation.

**Figure 2:**
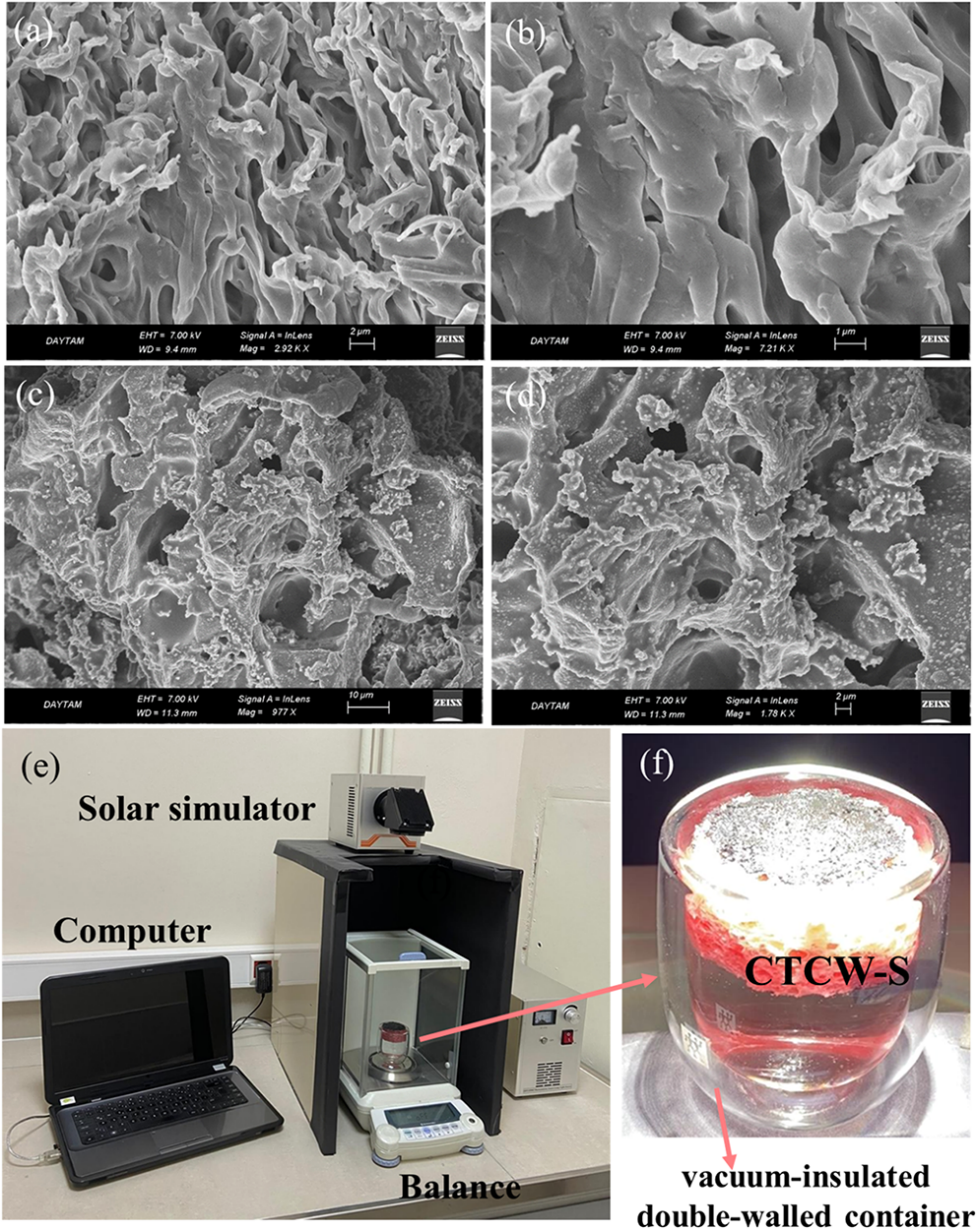
FESEM image of bare S (a, b) and CTCW-S (c, d) at different magnifications. Digital photograph of SSG device (e) and solar evaporator in double-walled glass container (f).

The effect of the amount of CTCW coated on the S surface was investigated for the preparation of CTCW-S. The evaporation rate of CTCW-Ss coated with different amounts of CTCW was determined, as shown in Figure S8a. The CTCW-Ss were named CTCW-3, CTCW-4, CTCW-5, and CTCW-6 for 300, 400, 500, and 600 mg CTCW, respectively. The results indicate that the evaporation rate increased with the amount of CTCW on the surface, but there is a slight decrement for CTCW-6 compared to CTCW-5. This can be attributed to the excess amount of CTCW that could not be wetted enough through the water transferred by the sponge and could not provide sufficient heat-vapor transfer. The effect of the sponge height on the preparation of CTCW-S was investigated, and the evaporation rates of sponge materials with different heights (0.5, 1.0, and 1.5 cm) were compared, covering the surfaces with 500 mg of CTCW. The results in Figure S8b show that the height of the sponge had almost no effect on the evaporation rate of the photothermal material. Therefore, the evaporator was prepared without changing the sponge height as purchased (1.5 cm) for easy preparation.

The SSG performance of CTCW-S was evaluated with the measurement system, as shown in Figure 2e. The system comprises a solar simulator, an electronic analytical balance for monitoring mass loss due to water evaporation, and a computer interface for recording and analyzing time-dependent evaporation rates. The temperature changes were monitored and recorded with a thermal camera. The experiments were carried out with an insulated double-walled container to prevent environmental heat conduction, including floating CTCW-S on water (Figure 2f).

The mass change of water for 60 min under 1 sun irradiation was determined to investigate the evaporation performance of CTCW-S in Figure 3a. The time-dependent evaporation rate of CTCW-S was determined to be 1.63 kg·m^–2^·h^–1^, which is approximately 5.2 times higher than that of pure water (0.31 kg·m^–2^·h^–1^) and about 2.5 times greater than that of bare S (0.65 kg·m^–2^·h^–1^). In Figure 3b, the temperatures of the samples (pure water, bare S, and CTCW-S) over time were examined during 100 s of irradiation. After 100 s, the surface temperature of pure water and bare S reached 24.9 and 29.4 °C, respectively, while that of CTCW-S rapidly increased during the first 85 s and stabilized at 55.2 °C. The maximum surface temperature of CTCW-S was higher than that of the bare S, indicating the strong photothermal conversion effect of the carbonized structure (CTCW). The surface temperature of pure water, bare S, and CTCW-S were monitored with an infrared camera over time under 1 solar irradiation (Figure 3c). The initial temperature of pure water was 15.8 °C. Noticeably, the surface temperature of CTCW-S sharply increased in the first 30 s and was recorded as 36.2, 42.6, 50.6, 47.0, and 55.4 °C after 30, 50, 70, and 90 s; in contrast, that of bare S exhibited low temperatures with less increment from 25.0 to 29.0 °C after 30 and 60 s, respectively. Digital photographs of CTCW-S and bare S as solar evaporators are shown in Figure S9. A cloud-like image consisting of micro water droplets was formed on the CTCW-S surface, indicating that the CTCW structure promoted rapid evaporation.

**Figure 3:**
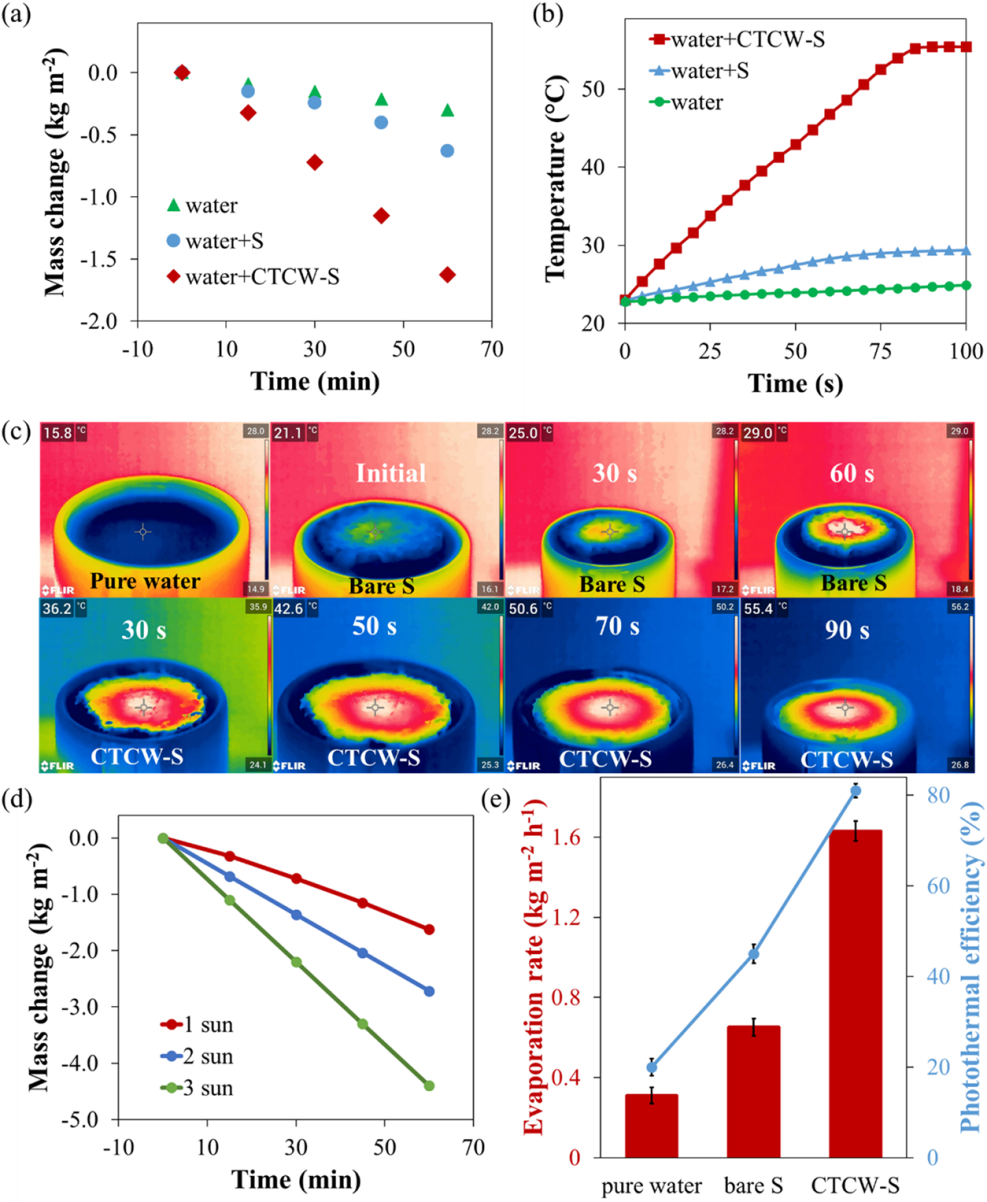
Time-dependent mass change (a) and surface temperature (b) of CTCW-S, bare S, and pure water under 1 sun irradiation. (c) Infrared images of pure water, bare S, and CTCW-S under different times. (d) Time-dependent mass change of CTCW-S under different solar irradiation. (e) Evaporation rate and photothermal efficiency of pure water, bare S, and CTCW-S under 1 sun irradiation.

When the light intensity was increased to 2 and 3 suns, the evaporation rate of the CTCW-S photothermal material increased correspondingly to 2.72 and 4.40 kg·m^–2^·h^–1^, respectively (Figure 3d). The photothermal efficiency of the samples was calculated using eqs 1–3, with detailed procedures provided in the Supporting Information. By subtracting the dark evaporation rate (0.21 kg·m^–2^·h^–1^) from the illumination-induced rate (1.63 kg·m^–2^·h^–1^), a net evaporation rate of 1.42 kg·m^–2^·h^–1^ was obtained for CTCW-S. The solar-to-vapor conversion efficiency calculated from eq S2 was 81%. For consistency, an illumination-induced evaporation rate of 1.63 kg·m^–2^·h^–1^ with a corresponding efficiency of 81% was adopted in this study.

Pure water, bare S, and CTCW-S exhibited photothermal efficiencies of 20, 45, and 81%, respectively (Figure 3e). CTCW-S exhibited a higher energy efficiency and water evaporation rates, highlighting its superior photothermal conversion performance. Moreover, the CTCW layer positioned on the surface of S acts as a thermal barrier, effectively minimizing heat dissipation and further enhancing the photothermal conversion efficiency. Preliminary calculations of energy payback time and overall energy efficiency, based on small-scale experiments and detailed in the Supporting Information, indicate that the fabrication energy of CTCW-S can be recovered through water evaporation, with both metrics expected to improve substantially in larger-scale applications.

In the SSG system, heat energy can be dissipated through three primary mechanisms: radiation, convection, and conduction, as illustrated in Figure S10. For CTCW-S, the radiation, convection, and conduction losses were calculated as 7.9, 4.8, and 6.3%, according to eqs S4–S6, respectively.^45^ Such low thermal losses demonstrated that CTCW on the S surface facilitates heat isolation. Compared with some reported systems,^25,46^ however, these losses are relatively higher, which is consistent with the lower photothermal efficiency of CTCW-S.

The evaporation performance of CTCW-S was further investigated for different solutions containing heavy metal (200 mg/L), acid (pH 2), alkaline (pH 12), dye (MB-1.0 g/L), and salt (NaCl-3.5 wt %) solutions. Figure 4 displays the photothermal efficiency, evaporation rate (Figure 4a), and mass change (Figure 4b) of CTCW-S in different solutions, and the results are close to those of pure water, demonstrating that our material has a comparable SSG performance in various solutions. Meanwhile, the surface temperature of CTCW-S on different solutions exhibited the range of ∼52–54 °C (Figure 4c), which is consistent with mass change. All of these findings revealed that CTCW-S is a potential solar evaporator in desalination and water purification applications.

**Figure 4:**
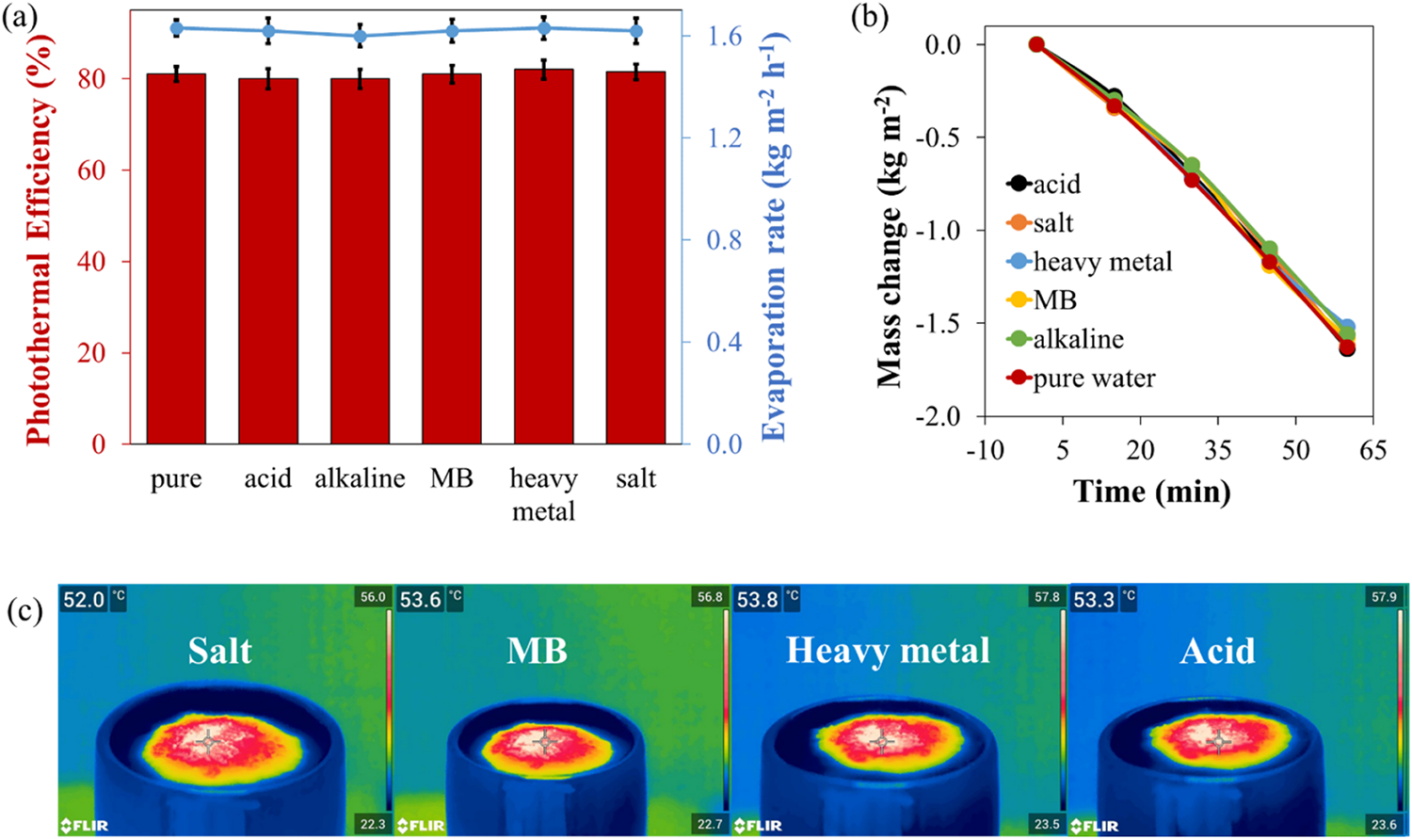
Photothermal efficiency and evaporation rate (a) and time-dependent mass change (b) of CTCW-S for pure water and solutions containing acid, alkaline, salt, heavy metal, and MB under 1 sun irradiation. (c) Infrared images of CTCW-S floating on solutions containing heavy metal, salt, acid, and MB.

To investigate the desalination performance of CTCW-S, the evaporation process was carried out until 10 mL of distilled water was obtained from about 20 mL of a simulated seawater (sea salt saline solution 3.5 wt %). The closed experimental setup shown in Figure S11 was used under 10 nm sunlight for the purification and desalination experiments. The ion contents of the solution before and after desalination were determined using ICP-MS. In the comparison of the ions in Figure 5a and Table S2, our evaporator exhibits a high desalination performance. In addition, to further evaluate the salt tolerance of CTCW-S, evaporation tests were performed with solutions containing different NaCl concentrations (5.0, 7.0, and 10.0 wt % NaCl), and the corresponding evaporation rates were recorded as 1.59, 1.48, and 1.31 kg m^–2^ h^–1^, respectively. These findings indicate that CTCW-S maintains an effective evaporation performance under saline conditions, highlighting its potential applicability for seawater desalination.

**Figure 5:**
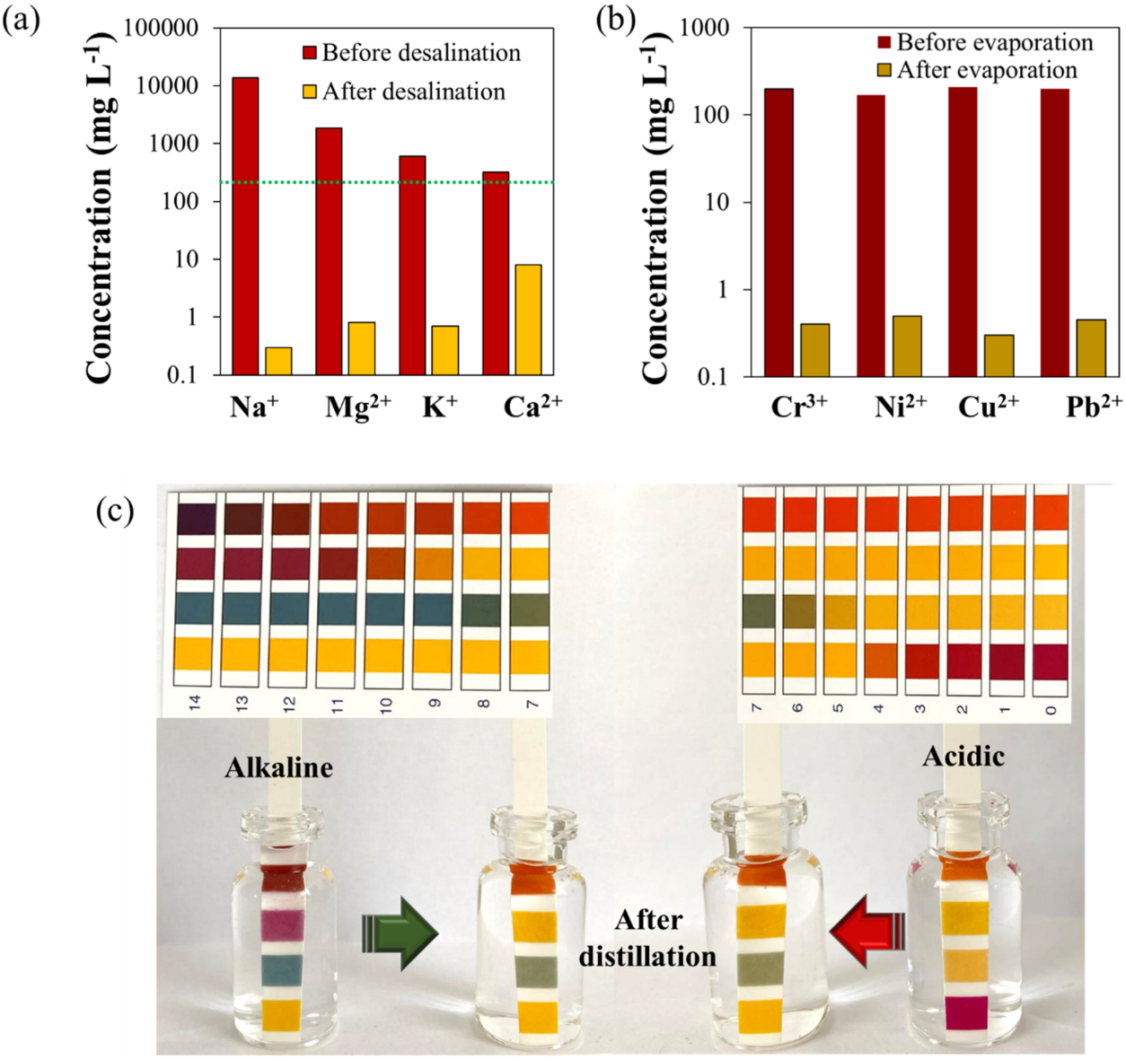
Comparison of (a) Ca^2+^, Mg^2+^, K^+^, and Na^+^ and concentrations before and after desalination and (b) the heavy metal ion concentration in the simulated wastewater before and after solar evaporation. (c) Digital photographs illustrating the pH values of acidic (pH 2) and alkaline (pH 12) solutions before and after solar purification using CTCW-S.

To test the desalination performance of CTCW-S under more challenging conditions, long-term experiments were conducted with a 5.0 wt % NaCl solution, which is more concentrated than typical seawater (3.5 wt %). Over a continuous 4-day operation, visible salt accumulation occurred on the surface of the evaporator (Figure S12). This deposition resulted in a 23% reduction in the evaporation rate, indicating that extended exposure to high salinity influences the performance. Despite this decline, CTCW-S maintained a notable evaporation capacity, demonstrating its promising potential for stable and repeated SSG operation even in highly saline environments.

To confirm the reliability of CTCW-S in seawater desalination, Black Sea seawater was used to conduct the actual test. The results in Table S3 confirm that our material has a broad application in seawater desalination. Noticeably, the amount of ions in distilled water after solar desalination of salty water and seawater is within the limits accepted by the World Health Organization (WHO) for drinkable water. In a similar study, the results of solar purification of a heavy metal solution are shown in Figure 5b and Table S4. As seen in Table S4, the initial concentrations of 200 ppm of Pb^2+^, 180 ppm of Ni^2+^, 175 ppm of Cu^2+^, and 210 ppm of Cr^3+^ were reduced to 0.4, 0.3, 0.4, and 0.5 ppm, respectively, after evaporation using CTCW-S. This high purification performance is likely due to the electrostatic attraction of metal ions to the CTCW material. On the other hand, the distilled water generated from acidic and alkaline solutions showed a pH value close to 7 (Figure 5c). All of these results further confirm that CTCW-S photothermal material has excellent solar desalination and purification performance.

To test the solar purification of the dye-containing solution with CTCW-S, an MB solution with a concentration of 1.0 g L^–1^ was placed in the prepared SSG setup, and the distillation process was applied. After irradiation for 2 h, the distillate water was examined through UV–vis absorption spectroscopy. Photographs of solar evaporation from initial periods to 1, 30, 60, 90, and 120 min are shown in Figure 6a–f. The distillate collected via the container tap (Figure 6g) was visually transparent (inset of Figure 6h), and its UV–vis spectrum exhibited the complete disappearance of MB absorption peaks, thereby confirming the efficacy of solar purification and highlighting the potential application of CTCW-S in wastewater treatment.

**Figure 6:**
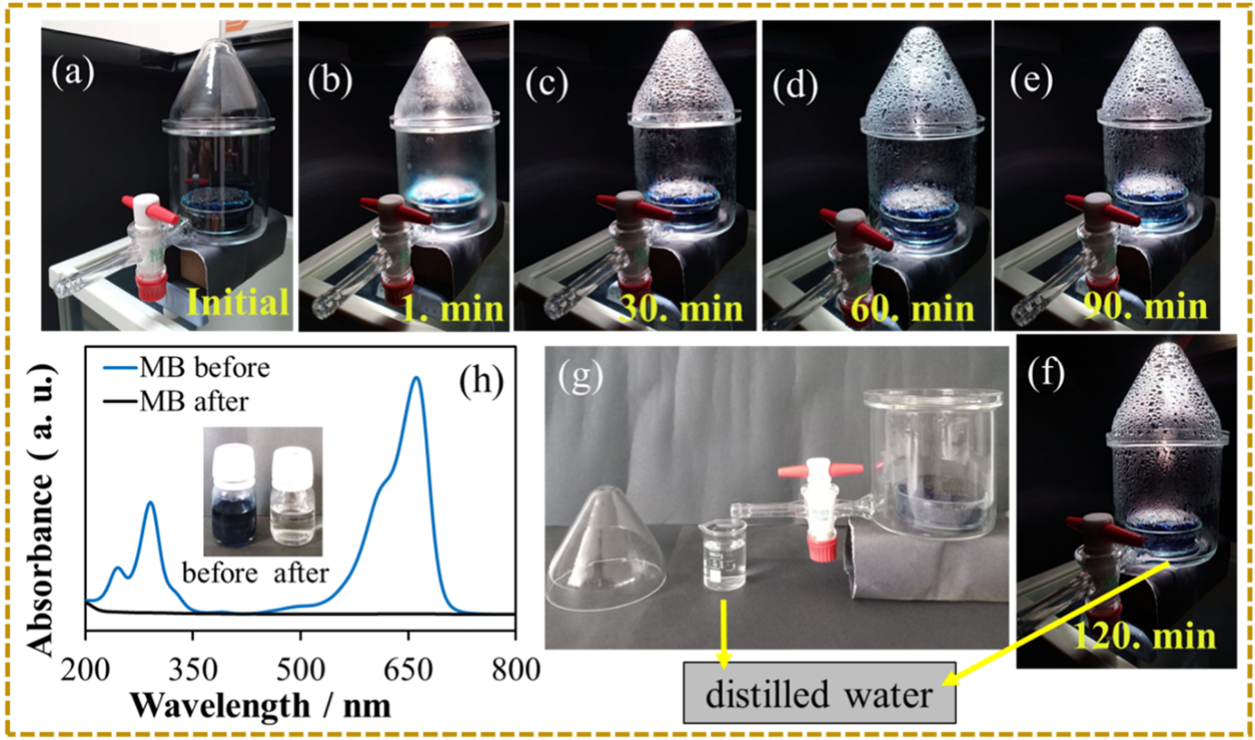
Digital photographs of solar purification with CTCW-S at different times (a–f) and distillate (g). (h) UV–vis spectra of MB before and after solar purification.

Outdoor experimental investigations were conducted in July under Erzurum climatic conditions, characterized by a relative humidity of 58%, an average temperature of 25 °C, sunrise at 05:00, and a sunset at 19:40. The changes in solar flux, the evaporation surface temperature, and mass were presented in Figure 7a. The results of the outdoor experiment with CTCW-S were quite close to those under 1 sun irradiation in the laboratory, revealing that our solar evaporator has great potential for practical application under natural sunlight.

**Figure 7:**
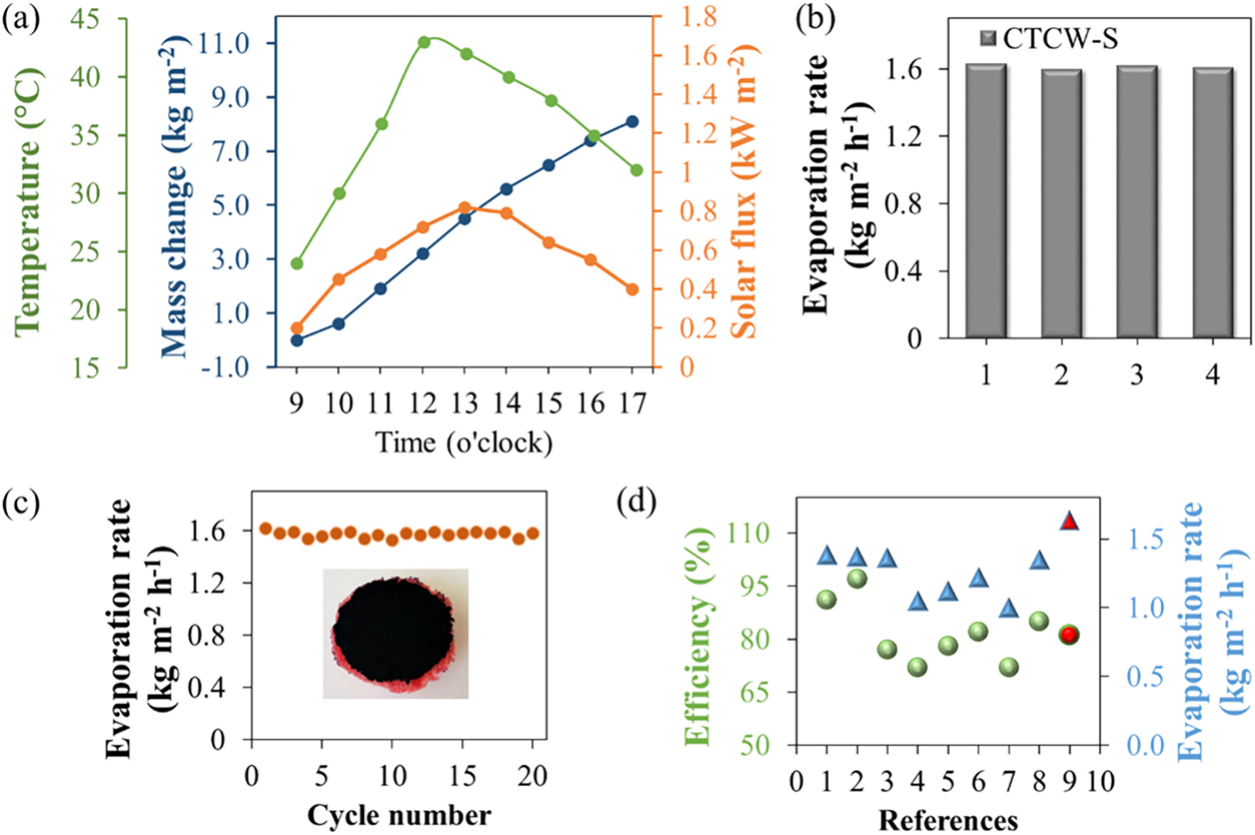
(a) Temperature, solar flux, and mass change under outdoor sunlight. (b) Reproducibility and (c) long-term SSG performance of CTCW-S. Inset: Photograph of CTCW-S after 20 cycles. (d) Comparison of the photothermal efficiency and evaporation rate with this work and other biomass-derived SSG materials.

To test the reproducibility of the material, four CTCW-Ss were prepared using the same method simultaneously and used as photothermal converters in the solar vapor generation system (Figure 7b). The similarity in evaporation rates among the materials indicates that the proposed preparation method reliably produces materials with consistent structural and chemical properties. To evaluate the long-term SSG performance stability of CTCW-S, a 3.5% NaCl solution was desalinated for 20 consecutive cycles under one solar irradiation. As shown in Figure 7c, the material exhibited an average evaporation rate of 1.57 kg·m^–2^ h^–1^, closely comparable to that of pure water, which was measured at 1.63 kg·m^–2^ h^–1^. Furthermore, no noticeable deformation was observed in CTCW-S after 20 cycles, as illustrated in the inset of Figure 7c. In addition, the SSG performance of CTCW-S was tested under strongly acidic and strongly basic conditions for 20 cycles, where only a slight decrease in evaporation efficiency was recorded (14.1% in the acidic medium and 13.6% in the basic medium), and the material retained its physical stability, as shown in Figure S13. These results demonstrate that CTCW-S possesses excellent cycling stability and remains suitable for repeated use even under harsh conditions such as strongly acidic and basic environments.

The extended cycling tests were conducted to evaluate the long-term performance retention of CTCW-S (Figure S14). The SSG performance was assessed over 50 successive evaporation cycles, yielding an average evaporation rate of 1.32 kg·m^–2^ h^–1^. After 50 cycles, the evaporation rate decreased by 19%, indicating a moderate but acceptable decline in performance under repeated operation. Postcycling characterization using FESEM revealed partial structural degradation, characterized by the slight enlargement of pre-existing pores, which is consistent with the observed performance loss (Figure S15a,b,d,e). Moreover, EDS spectra obtained before and after 50 cycles demonstrated that despite the physical deformation, the material retained nearly the same proportions of C and O elements, confirming that the intrinsic composition of CTCW-S remains stable during repeated operation (Figure S15c,d). These findings confirm that while some physical wear occurs after extensive cycling, CTCW-S maintains a considerable level of stability, demonstrating its potential for repeated and long-term solar steam generation applications.

Finally, the relevant performance of CTCW-S (ref 9 in Figure 7d) was compared with reported biomass-derived SSG materials^47–54^ (references numbered 1–8) in Figure 7d. CTCW-S exhibited superior steam generation rates and higher photothermal efficiency compared to these materials. Moreover, the material demonstrates strong potential for practical applications in wastewater treatment and solar desalination, owing to its cost-effectiveness, straightforward preparation process, and high performance. These attributes highlight its promising prospects and economic viability in the field of solar steam generation.

## 4 Conclusions

In this study, a novel and low-cost photothermal material (CTCW-S) was successfully fabricated by modifying a commercially available hydrophilic cellulosic sponge with carbonized Turkish coffee waste. The integration of the sponge’s inherent water absorption ability with the strong light-harvesting and efficient photothermal conversion properties of the biomass-derived carbonized material resulted in a highly efficient solar evaporator. The CTCW-S exhibited a remarkable water evaporation rate of 1.63 kg·m^–2^ h^–1^ under one solar illumination and achieved a photothermal conversion efficiency of 81%. Furthermore, the evaporator demonstrated stable, long-term water evaporation performance and effective solar purification capabilities, highlighting its durability and multifunctionality. These attributes, combined with the use of low-cost, waste-derived materials, indicate great economic benefits and confirm the significant potential of CTCW-S for scalable, practical applications in water purification and seawater desalination. Overall, this work introduces a sustainable and economically viable strategy for solar-driven desalination systems aimed at addressing the global freshwater shortage.

## Supplementary Material



## Notes

Schematic illustrations of material preparation; digital photographs of precursors and products; thermogravimetric, adsorption–desorption, and UV–vis–NIR analyses; solar evaporation and desalination performance data, including efficiency, stability, and ion content measurements; long-term cycling tests with morphological and elemental characterizations; and details of the experimental setup (PDF)
