# Supplementary material for: High-Efficiency Solar Steam Generation Using a Biochar-Modified Sponge Evaporator Derived from Turkish Coffee Waste
Source: ACS Omega. 2025 Oct 1;10(40):47397–406. doi: 10.1021/acsomega.5c06761 (PMC12529117; doi:10.1021/acsomega.5c06761)
Supplement: Supplementary file 1 [file ao5c06761_si_001.pdf]

Supporting Information

High-Efficiency Solar Steam Generation Using a Biochar-Modified Sponge Evaporator Derived from Turkish Coffee Waste

Şeyda Sefa[a], Kader Dağcı Kıranşan[a], and Ezgi Topçu[a*]

[a] Department of Chemistry, Science Faculty, Atatürk University, Erzurum, 25240, Türkiye
* Email: ezgitopcu@atauni.edu.tr

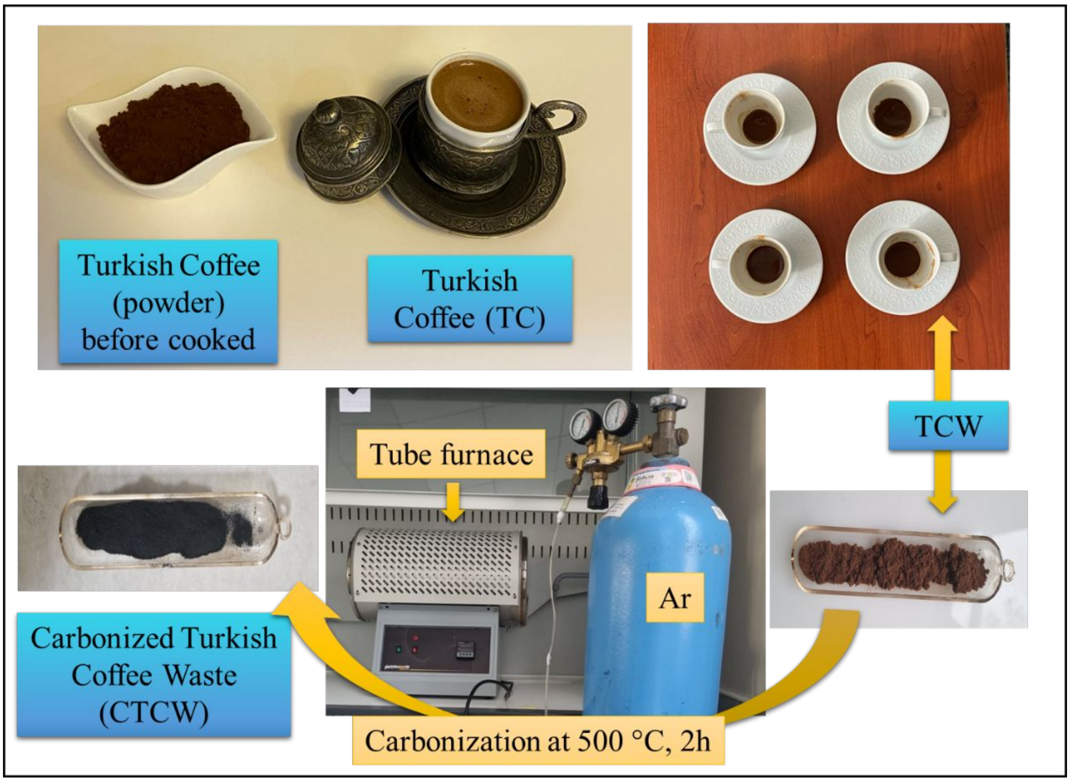

Figure S1. Schematic illustration of the preparation of CTCW.

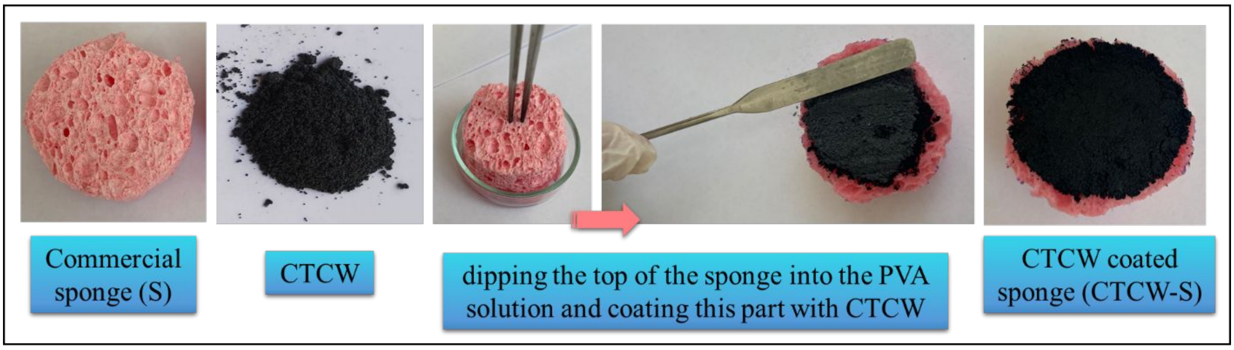

Figure S2. Schematic illustration of the preparation of CTCW-S.

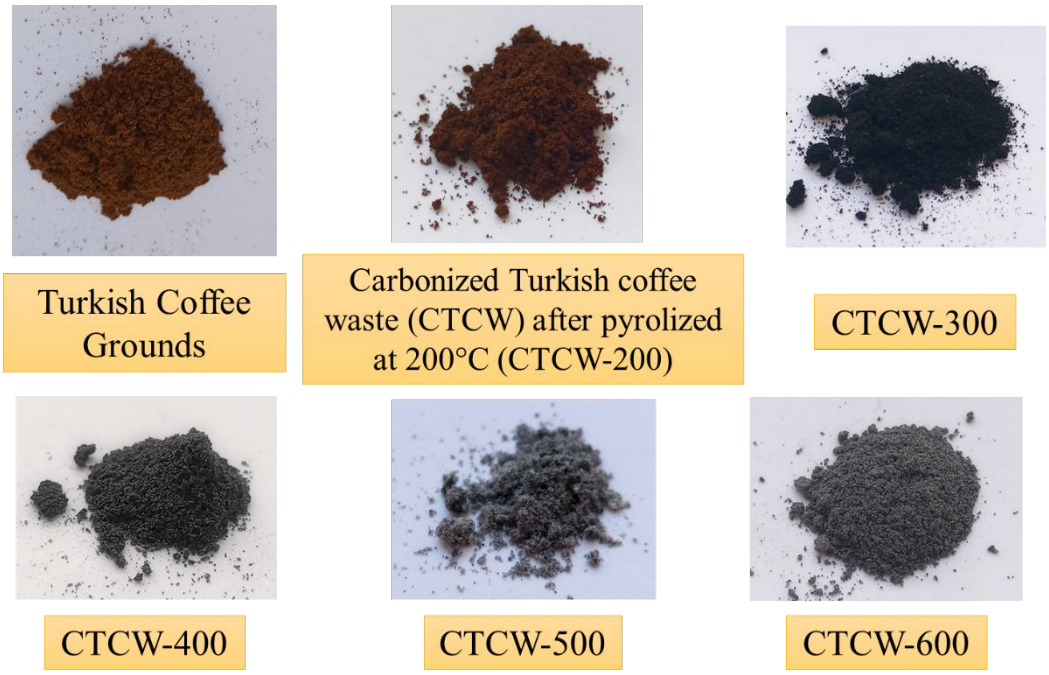

Figure S3. The digital photograph of Turkish coffee grounds and carbonized Turkish coffee wastes (CTCWs) at different temperatures.

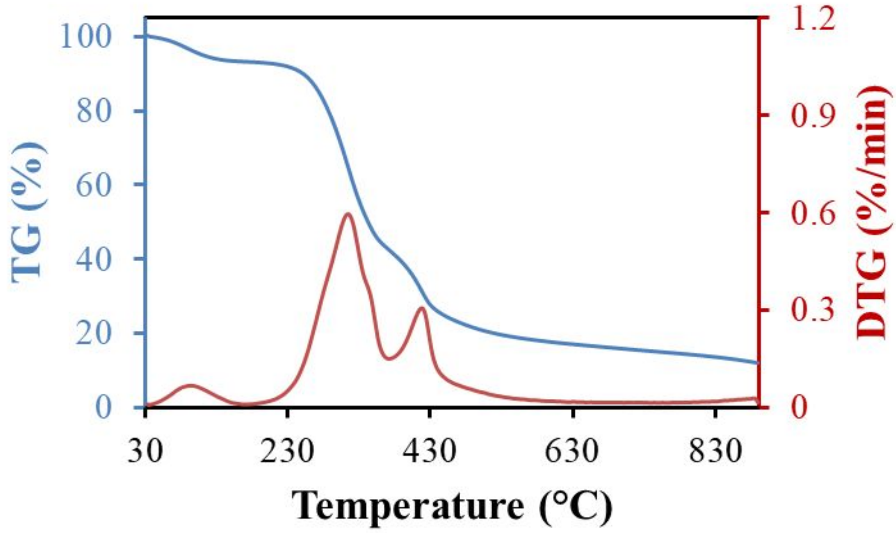

Figure S4. The thermogravimetric (TG) and derivative thermogravimetric (DTG) curves of TCW.

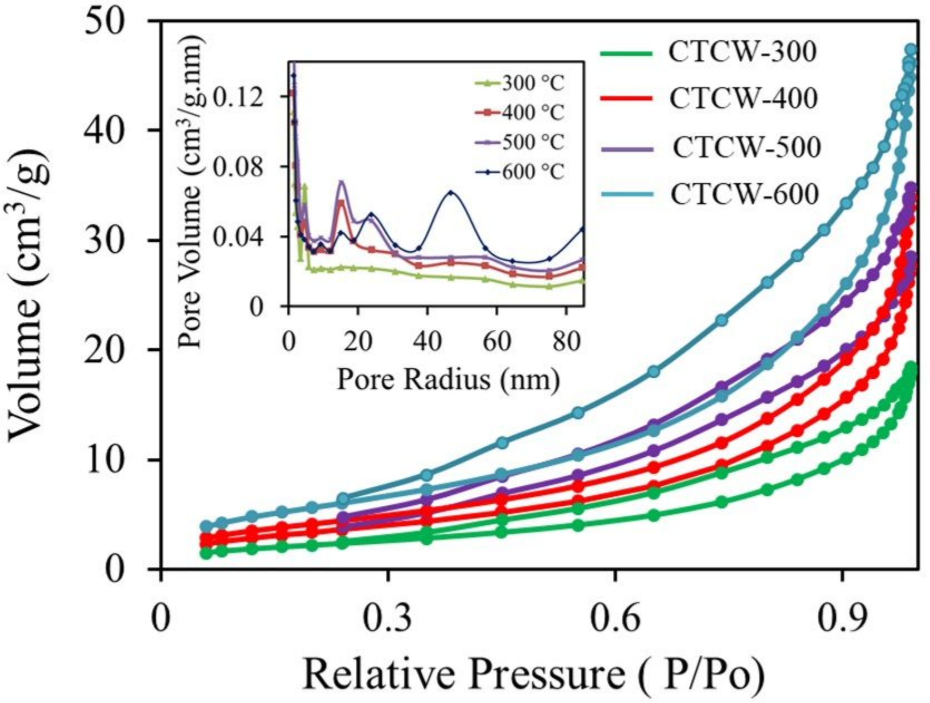

Figure S5. N_2 adsorption-desorption isotherms obtained at 77 K for CTCW-300, CTCW-400, CTCW-500, and CTCW-600. Inset: BJH pore size distributions of the samples.

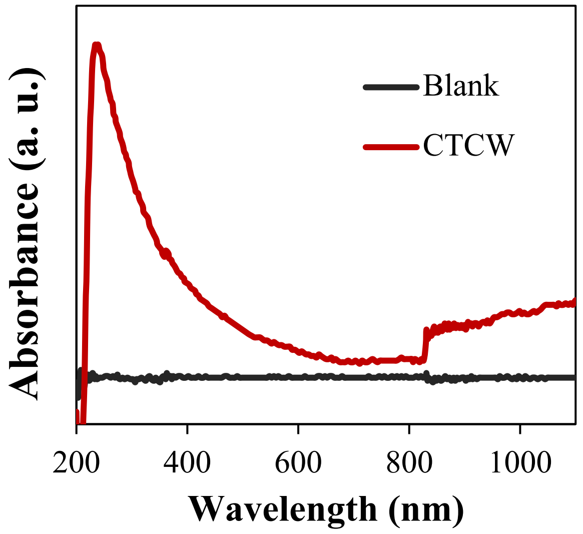

Figure S6. UV-VIS-NIR spectrum of CTCW.

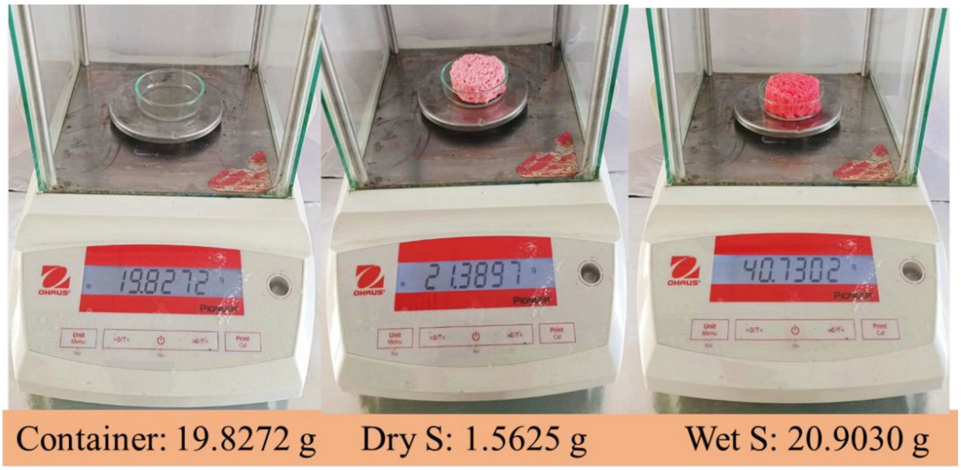

Figure S7. The dry and wet weight of S.

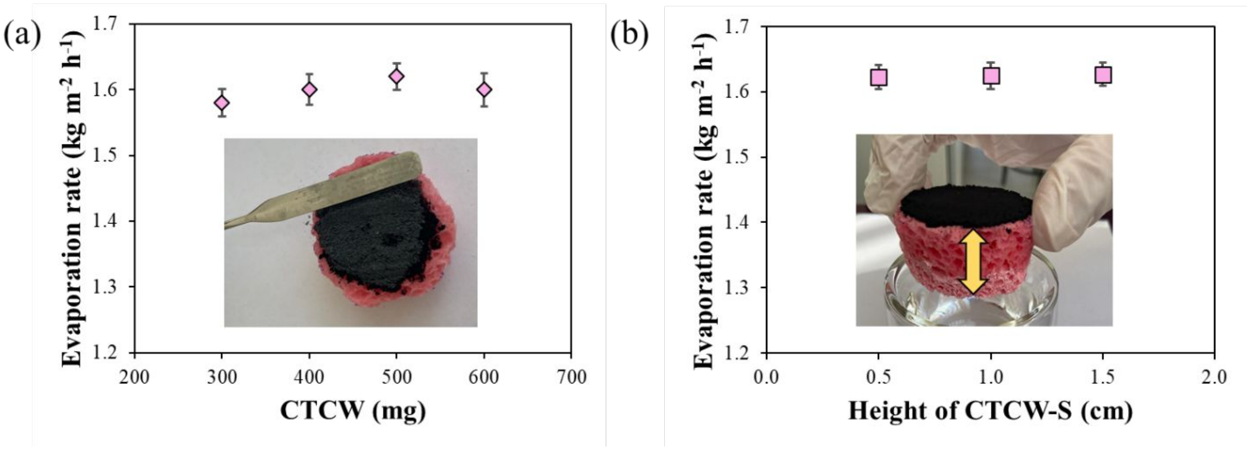

Figure S8. Evaporation rate of CTCW-S prepared with (a) different amounts of CTCW and (b) different heights of S.

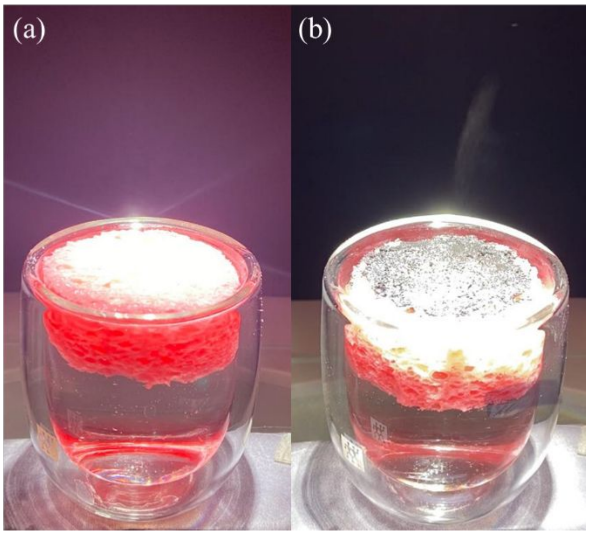

Figure S9. The photographs of bare S (a) and CTCW-S (b) at the 60. s of irradiation under solar irradiation.

Solar thermal conversion efficiency of CTCW-S:

The efficiency of solar thermal conversion is expressed as follows:

$$\eta = \frac{m.h_{LV}}{l} \qquad \text{(Equation S1)}$$

where η is the solar vapor conversion efficiency, m is the evaporation rate, h_{LV} is the sum of the total enthalpy of sensible heat and the phase change enthalpy of the liquid, and I is the energy of sunlight.[1]

To accurately evaluate the photothermal conversion efficiency (η) of CTCW-S, the net evaporation rate was obtained by subtracting the dark evaporation rate from the measured illumination-induced evaporation rate. Furthermore, the reduced latent heat of vaporization ($h_{\mathrm{LV,red}}$) was considered to account for the influence of the photothermal material on the water surface. The efficiency was then calculated using:

$$\eta = \dot{m}_{\mathrm{net}} \cdot h_{\mathrm{LV,red}} / I \qquad \text{(Equation S2)}$$

where $\dot{m}_{\mathrm{net}}$ is the net evaporation rate, $h_{\mathrm{LV,red}}$ is the reduced evaporation enthalpy, and I is the incident solar flux. Using this approach, the photothermal conversion efficiency of CTCW-S is determined to be ~81%, providing a realistic assessment of the system performance.[2]

When water molecules pass through in a porous medium, they are more likely to evaporate in

clusters rather than as single molecules.[3] Therefore, the evaporation enthalpy of water on the CTCW-S surface is lower than that of water.[4]

The enthalpies of water evaporation on the surfaces of sponge materials were calculated as follows:

$$m_w.h_w = m_e.h_e \qquad \text{(Equation S3)}$$

Here, m_w and m_e are the evaporation rates of the water without any material on it and the water on the material surface, respectively, and h_w and h_e are the enthalpy of water and the water on the surface, respectively [1]. Using Equation S2, the evaporation enthalpy for CTCW-S was calculated as and 1521±15 J g^{-1} (The evaporation enthalpy of water assumed as 2450 J g^{-1} at 25 °C). When S was modified with CTCW, the enthalpy of evaporation decreased,[5] attributed to the fact that CTCW can transfer heat energy more quickly and thus bulk water evaporation.

Table S1. Enthalpy of vaporization for S and CTCW-S.

**Material**	**Enthalpy of vaporization (h, J g^{-1})**
S	1926±16
CTCW-S	1521±15
The enthalpy of vaporization of water: 2450 J g^{-1} at 25 °C.<br>The results were calculated using the evaporation rates obtained under the same conditions on 5 different days.	

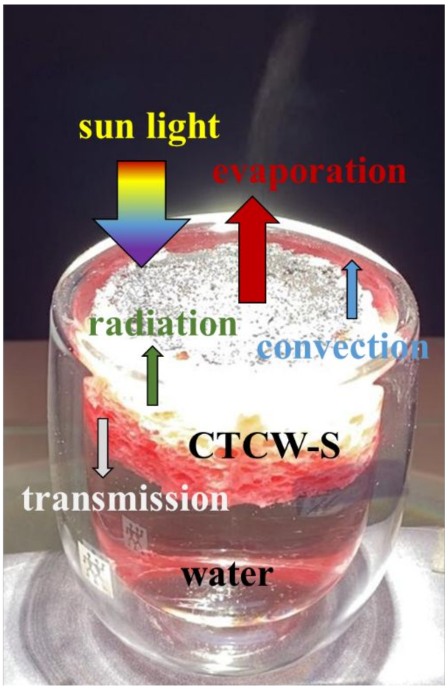

Figure S10. Energy balance and heat transfer diagram for CTCW-S photothermal material.

Heat losses for our SSG system:

The following was calculated for the heat losses.

i. Radiation.

Radiation loss was calculated using the Stefan−Boltzmann equation for 1 kW m^{-2} solar energy [6].

$$ER = \varepsilon A \sigma (T^4 - T\infty^4) \qquad \text{(Equation S4)}$$

where ER is the heat flux, ε is the emission (assumed as 0.97), A is the surface area (12.5 cm^2), σ is the Stefan−Boltzmann constant (5.67×10^{-8} W m^{-2} K^{-4}), T is the maximum temperature (max. 77 °C) and T∞ is the ambient temperature during the experiment (average 20 °C).

ii. Convection.

Convection loss was calculated by Newton's law of cooling [6].

$$Q = Ah(T - T\infty) \qquad \text{(Equation S5)}$$

where Q is the heat, h is the convection heat transfer coefficient, and A is the surface area (12.5 cm^2). The convection heat transfer coefficient is about 5 W m^{-2} K^{-1}.

iii. Conduction.

Conduction loss was calculated with the basic transmission law.[6]

$$Q = Cm\Delta T \qquad \text{(Equation S6)}$$

where Q is the heat, C is the specific heat capacity of water (4.2 J g^{-1} °C^{-1}), m is the weight of water (□30 g), and ΔT is how much the water temperature rises in 3600 seconds.

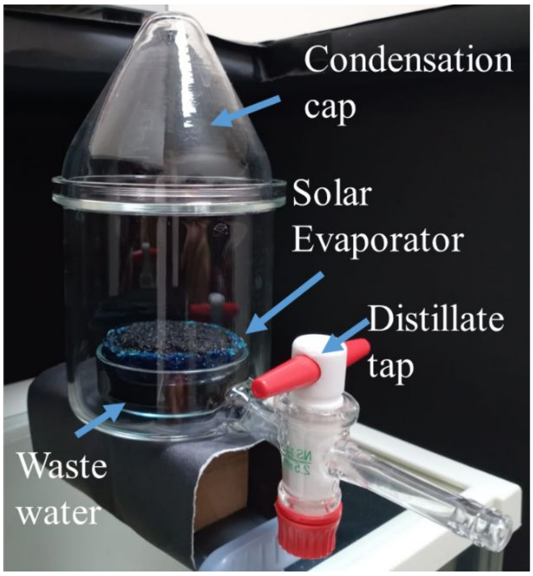

Figure S11. The experimental setup for solar desalination and purification test with CTCW-S.

Table S2. The ion contents after solar desalination of simulated seawater with CTCW-S.

**Na^+ (ppm)**	**K^+ (ppm)**	**Mg^{+2} (ppm)**	**Ca^{+2} (ppm)**
0.3	0.7	0.8	8.0
Initially, 13800 ppm Na^+, 600 ppm K^+, 1845 ppm Mg^{+2}, and 320 ppm Ca^{+2} were detected in seawater.			

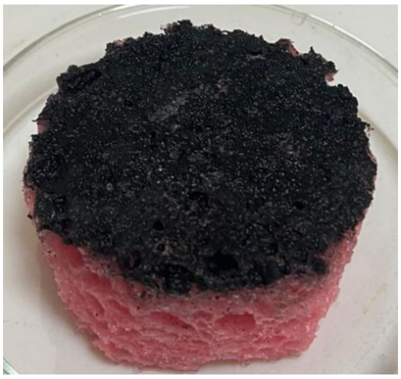

Figure S12. Photograph showing the amount of salt accumulated on the surface of CTCW-S after a continuous 4-day evaporation experiment with 5.0 wt% NaCl solution.

Table S3. The ion contents after solar desalination of Black Sea water with CTCW-S.

Samples	Na^{+} (ppm)	Mg^{+2} (ppm)	Ca^{+2} (ppm)
Black Sea seawater	15571	2112	400
Black Sea seawater after distillation	0.5	1.1	5.8

Table S4. The ion contents after solar purification of heavy metal solution with CTCW-S.

Ions of heavy metals			
Pb^{+2} (ppm)	Cu^{+2} (ppm)	Ni^{+2} (ppm)	Cr^{+3} (ppm)
0.4	0.3	0.5	0.4
Initially, 200 ppm Pb^{+2}, 180 ppm Ni^{+2}, 175 ppm Cu^{+2}, and 210 ppm Cr^{+3} were detected in heavy metal solution.			

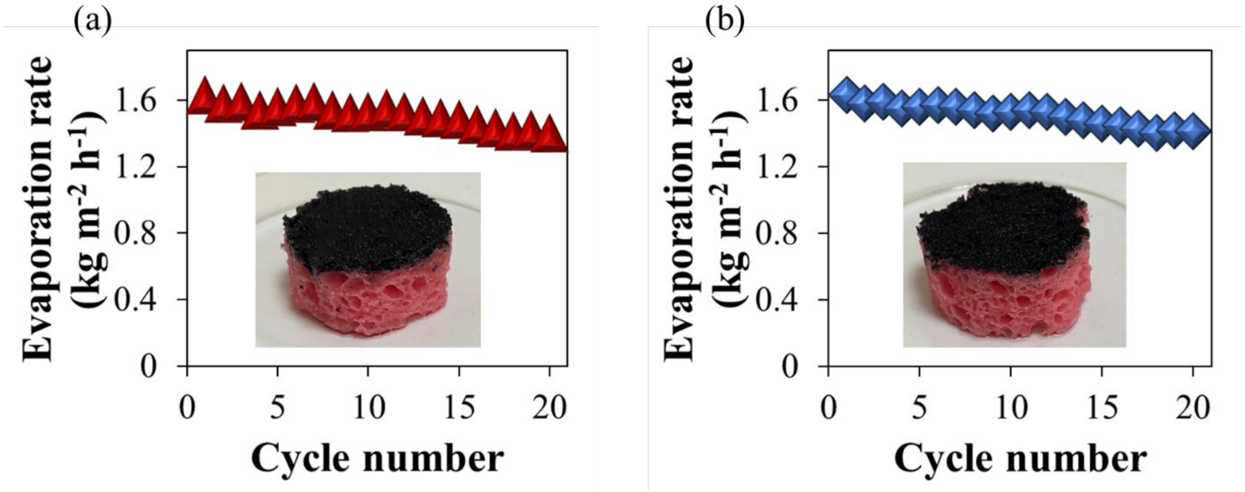

Figure S13. The long-term SSG performance of CTCW-S in (a) strongly acidic and (b) strongly basic medium. Inset: Photograph of CTCW-S after 20 cycles.

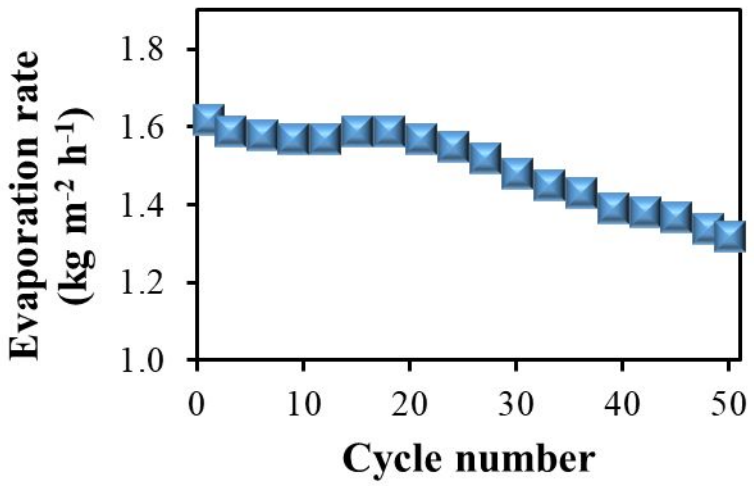

Figure S14. SSG performance of CTCW-S for 50 evaporation cycles.

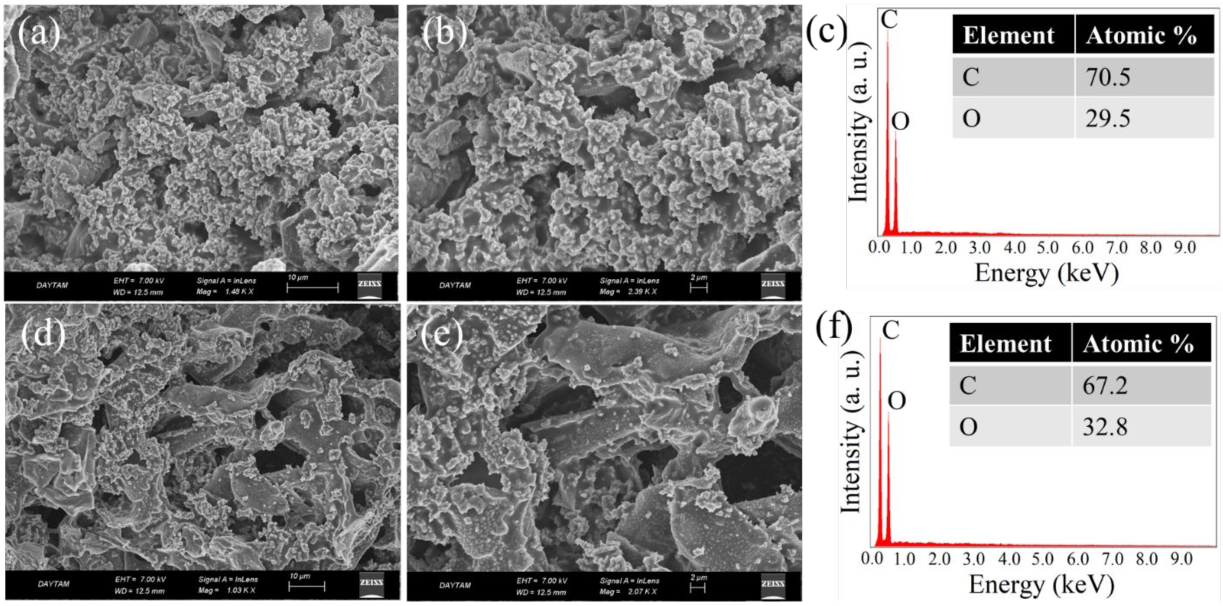

Figure S15. FESEM images of CTCW-S before (a, b) and after (d, e) 50 evaporation cycles, shown at different magnifications. EDS spectrum of CTCW-S before (c) and after (f) 50 cycles.

References

(1) Kim, C.; Shin, D.; Baitha, M. N.; Ryu, Y.; Urbas, A. M.; Park, W.; Kim, K. High-Efficiency Solar Vapor Generation Boosted by a Solar-Induced Updraft with Biomimetic 3D Structures. *ACS Appl. Mater. Interfaces* **2021**, *13* (25), 29602–29611. https://doi.org/10.1021/acsami.1c05883.

(2) Rathore, L. K.; Bera, A. Photo-Fenton-Active MIL-88A/CNT-Based PVA Hydrogel for Solar-Driven Water Evaporation and Simultaneous Volatile Organic Compound Removal. *ACS Appl. Mater. Interfaces* **2024**, *16* (33), 43670–43681. https://doi.org/10.1021/acsami.4c10367.

(3) Gong, F. (Frank); Li, H.; Wang, W.; Huang, J.; Xia, D. (David); Liao, J.; Wu, M.; Papavassiliou, D. V. Scalable, Eco-Friendly and Ultrafast Solar Steam Generators Based on One-Step Melamine-Derived Carbon Sponges toward Water Purification. *Nano Energy* **2019**, *58* (November 2018), 322–330. https://doi.org/10.1016/j.nanoen.2019.01.044.

(4) Zhao, F.; Zhou, X.; Shi, Y.; Qian, X.; Alexander, M.; Zhao, X.; Mendez, S.; Yang, R.; Qu, L.; Yu, G. Highly Efficient Solar Vapour Generation via Hierarchically Nanostructured Gels. *Nat. Nanotechnol.* **2018**, *13* (6), 489–495. https://doi.org/10.1038/s41565-018-0097-z.

(5) Erçarıkcı, E.; Topçu, E.; Kudaş, Z.; Aksu, Z.; Alanyalıoğlu, M.; Dağcı Kıranşan, K. An Effective Material for Solar Steam Generation Applications: Gradient Graphene Sponge. *Mater. Today Sustain.* **2024**, *26* (December 2023), 1–11. https://doi.org/10.1016/j.mtsust.2024.100701.

(6) Yang, Y.; Zhao, R.; Zhang, T.; Zhao, K.; Xiao, P.; Ma, Y.; Ajayan, P. M.; Shi, G.; Chen, Y. Graphene-Based Standalone Solar Energy Converter for Water Desalination and Purification. *ACS Nano* **2018**, *12* (1), 829–835. https://doi.org/10.1021/acsnano.7b08196.
